# Semiochemical Production and Laboratory Behavior Response of the Brown Marmorated Stink Bug, *Halyomorpha Halys*

**DOI:** 10.1371/journal.pone.0140876

**Published:** 2015-11-03

**Authors:** Christina Harris, Sitra Abubeker, Mengmeng Yu, Tracy Leskey, Aijun Zhang

**Affiliations:** 1 Invasive Insect Biocontrol and Behavior Laboratory, Agricultural Research Service, United States Department of Agriculture, Beltsville, MD, 20705, United States of America; 2 Department of Entomology, Virginia Tech University, Blacksburg, VA, 24060, United States of America; 3 Appalachian Fruit Research Station, Agricultural Research Service, United States Department of Agriculture, Kearneysville, WV, 25430, United States of America; Rutgers University, UNITED STATES

## Abstract

**Background:**

The brown marmorated stink bug (BMSB) is an exotic insect pest that was first recognized in the United States in 2001. As of today, it has been found in more than 42 states. BMSB has a very broad host plant range and damage to crops in mid-Atlantic States has reached a critical level. A reliable and accurate tool for infestation detection and population monitoring is urgently needed to provide better and more timely interventions. Pheromones produced by male BMSB have been previously identified and are currently used in BMSB infestation detection. However, the conditions affecting BMSB production of these pheromones were unknown.

**Methodology/Principal Findings:**

In this study, we collected headspace volatiles from male BMSB under laboratory conditions, measured the temporal patterns of release of these pheromones, and assayed the attractiveness to conspecifics. In addition to the pheromone components, tridecane (C13) and *E*-2-decenal (an alarm compound) were observed in headspace collections of males, as well as in females and nymphs. Exposure of pheromone-emitting adult males to synthetic C13 greatly reduced pheromone emission.

**Conclusions/Significance:**

This information should lead to a better understanding of the biology, physiology, and chemical ecology of BMSB, which will help scientists and growers develop more efficient strategies based on natural products to manage BMSB population, therefore, reducing pesticide usage and protecting the crops from BMSB damage.

## Introduction

*Halyomorpha halys*, or the brown marmorated stink bug, is an invasive species of true bugs (Hemiptera: Pentatomidae) that has exhibited tremendous population growth in the Mid-Atlantic region of the U.S. since recognition of its introduction in 2001 [1–3]. Damage to stone and pome fruit caused by piercing/sucking feeding from this insect can be significant throughout the growing season [4,5]. Because *H*. *halys* has become well-established, populations have exerted tremendous season-long pest pressure complicating management for tree fruit growers and leading to devastating levels of fruit injury in the Mid-Atlantic region, with some growers losing their entire crop [3]. Currently, insecticide programs are being developed, but attempts to manage this insect under field conditions were proven fairly ineffective because applied materials did not ultimately result in mortality of individuals or decreased adult populations. Substantial knockdown and recovery from pyrethroid-based insecticides [6,7] and neonicotinoids [6] in the laboratory and field have been documented.

Pentatomids have been shown to attract conspecifics via male-produced pheromones [8,9] and acoustic signaling [10,11]. Attempts to monitor *H*. *halys* using the cross-attractant (*E*,*E*,*Z*)-2-4-6-decatrienoate from *Plautia stali* Scott have been shown to be moderately effective [12]. Recently, a *H*. *halys* male-produced pheromone blend, *trans and cis*-zingiberenol epoxides [(3S,6S,7R,10S)-10,11-epoxy-1-bisabolen-3-ol and (3R,6S,7R,10S)-10,11-epoxy-1-bisabolen-3-ol)], was identified and synthesized with demonstrated applicability in field settings for capturing conspecifics [13].

In the laboratory condition, we found that males *H*. *halys* begin emitting pheromone (~ 13 days old) as the number of male conspecifics decreases, suggesting that it is an aggregation pheromone. Two other compounds are also emitted from males as major components: tridecane (C13) and *E*-2-decenal (the aldehyde associated with alarm pheromone). Headspace airborne collections from females and nymphs show that C13 and *E*-2-decenal are also present, but not the male-specific pheromone blend emitted from single males.

Here, we explore the temporal parameters of the male-produced pheromone, C13, and *E*-2-decenal, and the resulting attractiveness of the matured male to conspecifics. We found that adult males begin releasing the pheromone 9–18 days post-imaginal ecdysis and will continue releasing this pheromone for up to 100 days in ~3-day cyclic bursts. C13 and *E*-2-decenal are released in a less cyclic pattern. Pheromone emission from single males is higher from 9 a.m.-3 p.m., while C13 and *E*-2-decenal emission is higher in the evening and overnight. Nymphs produced similar amounts of C13 to males, while females produced much less. Conversely, nymphs produced more *E*-2-decenal than adult males of females.

Our studies show that the amount of C13 increases and pheromone decreases with *H*. *halys* density. We hypothesized that synthetic C13 would reduce pheromone emission from *H*. *halys* males. This was confirmed in our laboratory experiment. We found that addition of synthetic C13, but not C13 and *E*-2-decenal, effectively reduces the presence of male-produced pheromones. Lastly, we used Y-tube bioassays to determine which sexes and age groups were most attracted to pheromone producing males.

## Materials and Methods

### Field Permit

Specific permission is not required for collection of BMSB annually in USDA, ARS, Beltsville Agricultural Research Center, Beltsville, MD. The land was owned by our employer, USDA. This collection will not involve endangered or protected species.

### Insects

The *H*. *halys* colony was established in 2007 from adults collected in Allentown, PA, and reared on a diet of organic green beans and seeds (2:1 sunflower: buckwheat seed). Insects were maintained in Thermo Forma chambers (Thermo Fisher Scientific^®^) at 25°C and 72% relative humidity with 16:8 hour light: dark cycle. The colony was replenished with ~20 field-collected bugs annually that were collected in USDA, ARS, Beltsville Agricultural Research Center, Beltsville, MD.

### Semiochemical Collection and Analysis

*H*. *halys* adults used in airborne collections were moved into separate all-male or female rearing containers two days post-imaginal ecdysis, at which point they were held until transferred to aeration containers under laboratory conditions based on methods previously developed for collecting volatiles for other insect species [14]. *H*. *halys* were placed individually (n = 116 males, n = 15 single females) or in groups (n = 89 grouped males with 6-29/group, n = 8 grouped females with 8-14/group, n = 59 third-stage nymphs with 6–20 per group in same age) in 1-liter, 4-necked glass containers with green beans and water. Volatile airborne collections were started using 5–11 day-old same sex virgin adults or 3^rd^ stage nymphs, and conducted in 24-hour increments for up to three months. Humidified air was drawn into the container through 6–14 mesh activated charcoal (Fisher Scientific®, Pittsburgh, PA), and out through two traps (15 cm x 1.5-cm OD) containing Super Q (200 mg each; Alltech Associates, Inc.®, Deerfield, IL) by vacuum (~1 liter/min). Insects were fed organic green beans (replaced every 2–3 days) and provided water tubes with cotton balls, and aerated continuously for 20 to 100 days depending on insect living conditions at room temperature (23–25 ^0^C) and 16L: 8D photoperiod. The adsorbent traps were changed every day (some of them in 3 days for weekend) and eluted with methylene chloride (0.5 ml/each sample). The eluents were stored in –30 ^0^C freezer until analyses.

Aeration samples were analyzed using a 30m x 0.25mm x 0.25μm inner diameter non-polar methyl silicone HP-5 capillary column (J&W Scientific Inc^®^) on an Agilent 6890 gas chromatography (GC) equipped with an HP auto-sampler in splitless mode (1.4 ml/min hydrogen carrier). Oven temperature was programmed at 40°C for 2 min, then to 280°C at 15°C/min and held for 7min. A hydrocarbon, tetradecane (10 ng/μL), was used as external standard for quantitative analysis. Gas chromatography-mass spectrometry (GC-MS) analyses of semiochemicals were conducted on an HP 6890 GC equipped a HP 5973 Mass Selective Detector using same column and oven temperature programs as GC, but with helium as carrier gas (1.4 ml/min). A 70 eV electron beam was employed for sample ionization [15]. Synthetic chemical standards, tridecane, tetradecane, and *E*-2-decenal were purchased from Sigma Aldrich (St. Louis, MO) and Bedoukian Research, Inc. (Danbury, CT).

### Pheromone Inhibition Tests

Male airborne collections were analyzed daily for pheromone emission as described above. When 3000 to 5000 ng/day was obtained and consistent for 2–3 days, a permeable polyethylene vial (26 mm x 8 mm x 1.5 mm thick, Just Plastic Ltd., Norwich, UK) containing 20 μL pure pheromone antagonist compound (C13 or C13+*E*-2-decenal in 10:1 ratio) with closed cap was placed into the aeration chamber for 24 hours. The volatile collection and analysis were continued for at least three days after the 24 hour treatment period. In a second application, 20–30 μL C13 or 20 μL C13+ *E*-2-decenal (10:1 ratio) was reapplied to the same chamber one week after the initial antagonist test to check for repeatability.

### Y-tube Bioassays

Individuals used in Y-tube assays were either classified as young adults (2–7 days post-adult emergence), old adults (13–23 days post-adult emergence), or 3^rd^ stage nymphs (13–20 days post-egg eclosion). Individuals from each group were tested in a Y-tube olfactometer for their attraction to live males. Y-tube bioassays were conducted from September-November of 2011 between 8 a.m. and 4 p.m., in a fume hood lined with black paper and covered with black curtain to prevent external visual stimuli. The Y-tube measured 30 cm for the straight tube and 15 cm for either arm, with an 8 cm diameter. At the end of each arm and the entry port for tested subjects was a modified 1L rounded flask [14]. Ambient air was pulled through an activated charcoal filter at 1 L/min and aerated with distilled water before being split through each arm. The fume hood was illuminated by a single 60 watt light bulb with a light intensity of 800 LUX, placed 50 cm above the fork in the Y-tube, with 26–28°C temperature and 50% RH. Plume structure and wind velocity were checked using incense smoke prior to the first assay.

Prior to being tested, insects were moved from Thermo Forma cages to individual 15 x 100 mm Petri dishes for one hour before being placed in the Y-tube. The Y-tube was rinsed with water and acetone after six bioassays had been conducted and if a new age group was to be evaluated. Individual insects were introduced into the release chamber and permitted to walk into either flask at the end of an arm (odor or control). If an insect did not respond within 10 minutes, it was recorded as “non-responder”. Individuals were used only once in these trials.

Attraction of nymphs, old adult males and females, and young adult males and females was observed toward live pheromone-producing males. Daily GC monitoring of samples from male aeration chambers were used to verify that males used as “Live Male” treatments in the Y-tube were actively releasing pheromone, and male lures were moved into the Y-tube 30 minutes prior to release of the test subject. A blank (empty) Y-tube was used to test *H*. *halys* movement in the assay chamber when no odors were present to ensure no bias in position.

### Statistical Analyses

The average and standard error of pheromone, C13, and *E*-2-decenal emission was calculated for single and grouped males, females, and nymphs by averaging the average/day of each aeration chamber. The start age that males began producing pheromone, C13, and *E*-2-decenal was calculated using average and standard error of individual males with statistics run as above. The start age by year was also calculated. Male-produced semiochemicals occurred in cyclic peaks, thus we calculated the average time in days between odor bursts, and tested whether this varied significantly by individual male using ANOVA (JMP^®^).

The effect of male density on compound emission was graphed using log-transformed data and repeated-measures ANOVA was used to test the effect on each compound. The relationship between C13 and pheromone was analyzed via ANOVA pairwise comparisons. Average and standard error of compounds was calculated for single males by time of day. Time periods were standardized for number of hours collected and ANOVA was used to test for effects. Tukey HSD comparisons tested all compounds and times combined.

Y-tube bioassays were analyzed for each age group and treatment using Likelihood Ratio tests (JMP^®^), with the likelihood of entering either arm set at 50% and the confidence interval set at 95%. Difference in movement towards live males was analyzed for each age/sex using Nominal Logistic Regression.

## Results

### Patterns in Pheromone Emission

Single adult males produced more male-specific pheromone than groups of males (Table 1) while groups of males produced more C13 than single males. *E*-2-decenal was not a major proportion of the male odor blend. Nymphs and females did not produce pheromone. Single and grouped females and 3^rd^ stage nymph groups produced similar amounts of C13 and *E*-2-decanal. Grouped males released 10-fold more C13 than nymphs, and 100-fold more than females. Nymphs released 100-fold more *E*-2-decenal than males and grouped females and 10-fold more than single females (n = 116 single males; n = 89 grouped males with 6-29/group; n = 15 single females; n = 8 grouped females with 8-14/group; n = 59 third-stage nymphs with 6–20 per group).

**Table 1:** Compounds emitted by males, nymphs, and females (ng/day).

	Pheromone	C13	*E*-2-decenal
Male (single) n = 116	843 ± 89	2,884 ± 742	99 ± 29
Male (6–29 in group) n = 89	74 ± 44	24,088 ± 9907	18 ± 7
Nymphs (6–20 3^rd^ stage), n = 59	0 ± 0	4,518 ± 797	3,009 ± 305
Female (single), n = 15	0 ± 0	827 ± 297	422 ± 288
Females (8–14 in group), n = 8	0 ± 0	133 ± 97	61 ± 17

The average and standard error of start age of the male-produced pheromone was 12.71 days ± 1.00 (n = 28). Males were placed in chambers at various ages, from 5–12 days into adult stage, but this did not affect the start age of pheromone emission. ANOVA: Age produced = Age started, year. R^2^-.258. F_4,27_−2.00, p = 0.128. Age started (days): F-0.707, p = 0.409, Year: F-3.808, p = 0.633, n = 28. Start age (days) for pheromone in 2011: 13.29 ± 0.618 (n = 24), 2012: 11.37 ± 1.117 (n = 8); C13 2011: 13.75 ± 1.482 (n = 24), 2012: 9.00 ± 1.376 (n = 8); and *E*-2-decenal in 2011: 20 ± 2.540 (n = 15), *E*-2-decenal in 2012: 13.5 ± 3.202 (n = 4).

**Fig 1:**
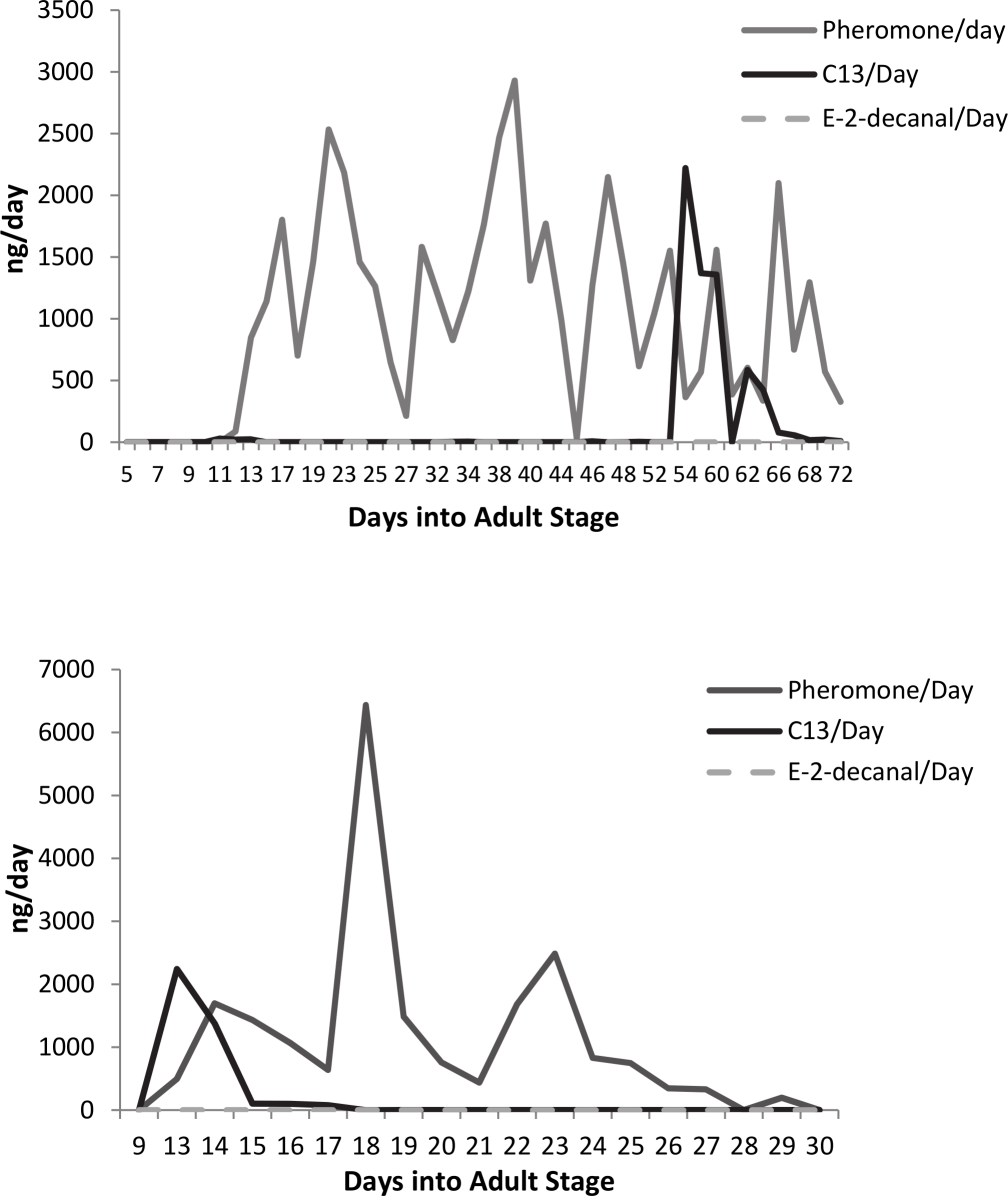
Pheromone/C13/*E*-2-decenal cycling for single male. The average ± SE start age of pheromone emission: 12.71 days ± 1.00 (n = 28). Start age of pheromone emission did not significantly vary by the age that males were placed in the jar (ages 5–12 days, n = 28). Pheromone emission was peaked every 3.04 ± 0.08 days, with an average of 3.33 ± 0.27 peaks per male. C13 and *E*-2-decenal emissions lasted longer 6.90 ± 0.53 days for C13, and 6.64 ± 0.80 days for *E*-2-decenal. C13 and *E*-2-decenal usually do not show repeated peaking trend, with average number of peaks being 1.91 ± 0.16 for C13 and 0.64 ± 0.16 for *E*-2-decenal (n = 84 males). (A) Example of cyclic pheromone and C13 peaks for a male held singly and placed in aeration chamber five days into the adult stage. (B) A male removed from an all-male holding cage and placed in an aeration chamber on day 9.

Cyclic patterns in emission of compounds were quantified using the numbers of days between peaks (Fig 1). Pheromone emission peaked roughly every three days. C13 and *E*-2-decenal did not frequently show a repeated peaking trend, rather there were one or two bursts per airborne collection. Fig 1A is an example of cyclic pheromone and random C13 peaks for a male held singly and moved to an aeration chamber five days into the adult stage. Fig 1B shows a male removed from an all-male holding cage and placed in an aeration chamber on day 9. This initial peak of C13 was frequently observed for the first 1–2 days of airborne collections if the male was not held singly. Average days between compound emission peaks are more frequent for bursts of pheromone emission relative to C13 and *E*-2-decenal. Average ± SE of days between pheromone peaks: 3.04 ± 0.08 days (n = 68 single males, 261 peaks); C13: 6.90 ± 0.53 days (n = 59 single males, 140 peaks); *E*-2-decenal: 6.64 ± 0.80 days (n = 53 single males, 140 peaks). Repeated Measures ANOVA: Square Root (Days Between Peaks) = Subject (Compound), Compound, Month: R^2^ = 0.53; F_113,453_−3.54, p <0.0001. Effect tests: Month F-0.675, p = 0.743; Compound F-44.086, p <0.0001. The average number of peaks per single male subject was: Pheromone 3.33 ± 0.27; C13 1.91 ± 0.16; *E*-2-decenal 0.64 ± 0.16. n = 84, Repeated Measures ANOVA: Square-root (Average number of peaks) = Subject (Compound), Compound: R^2^ = 0.22; F_2,249_−34.556, p <0.0001.

**Fig 2:**
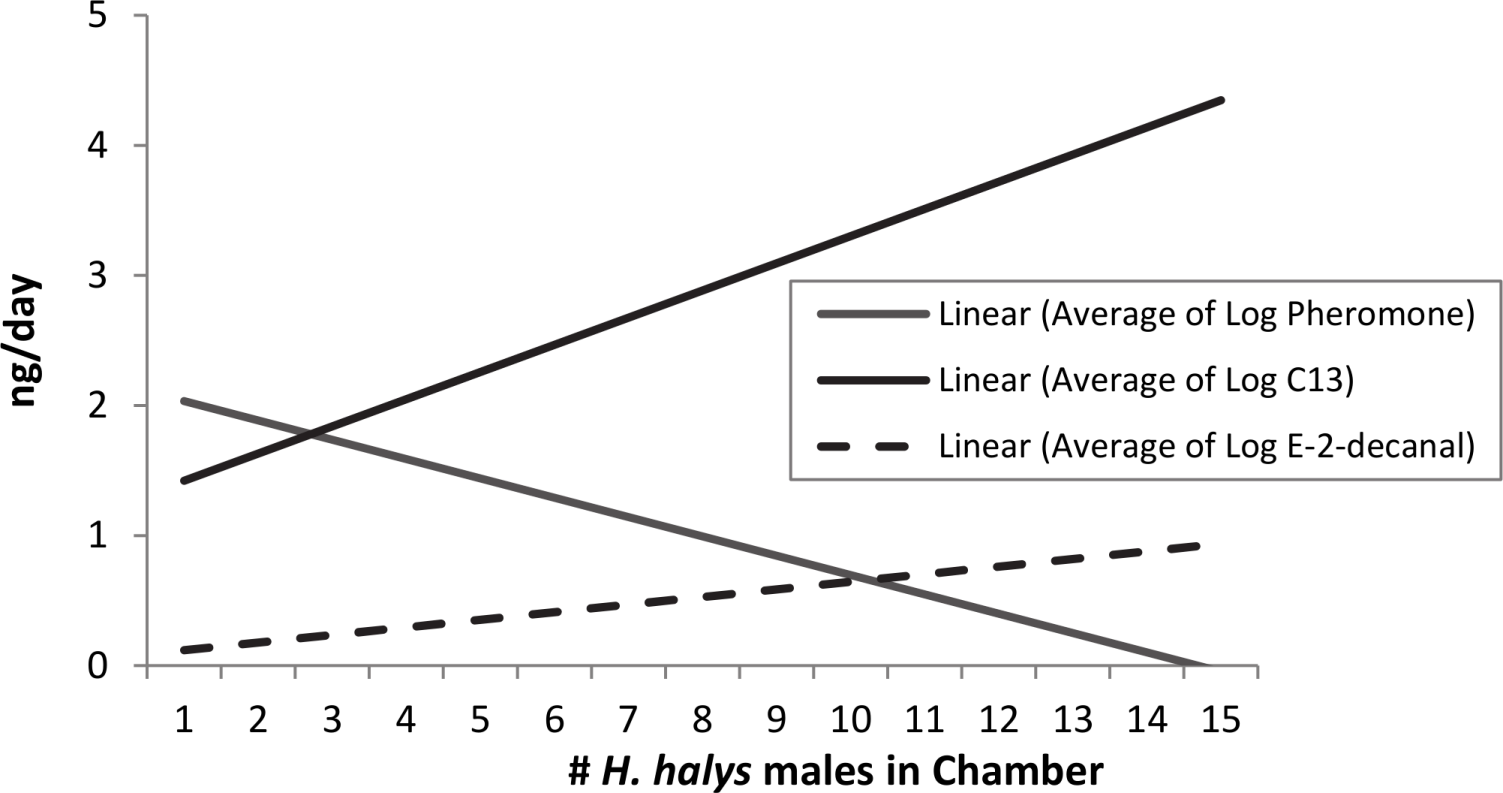
The amount of pheromone, C13, and *E*-2-decenal varies by number of male *H*. *halys* adults in chamber. This data shows 24-hour airborne collections from 2011–2012 on 13–90 day-old adults, with log-transformed data using repeated measures ANOVA for Compound = subject, # males (subject). Pheromone F_1,71_−6.634; p < 0.0001; C13 F_1,71_−5.701; p = 0.001; *E*-2-decenal F_1,71_−1.617; p = 0.003. Regression equations: Pheromone = -0.17x + 2.52; C13 = 0.22x + 1.15; *E*-2-decenal = -0.773x + 0.290. The relationship between C13 and the male-specific compounds was analyzed via ANOVA using pairwise correlations. Pairwise correlations, p < 0.0001. The C13 and male specific compounds interaction overlaps at 2.50 males.

The amount of pheromone, C13, and *E*-2-decenal present varied by number of male *H*. *halys* adults in chamber (Fig 2). These data shows 24-hour airborne collections from 2011–2012 on 13–90 day-old adults, with log-transformed data using repeated measures ANOVA for Compound = subject, # males (subject). Pheromone F_1,71_−6.634; p < 0.0001; C13 F_1,71_−5.701; p = 0.001; *E*-2-decenal F_1,71_−1.617; p = 0.003. Regression equations: Pheromone = -0.17x + 2.52; C13 = 0.22x + 1.15; *E*-2-decenal = -0.773x + 0.290. The relationship between C13 and the male-specific compounds was analyzed via ANOVA using pairwise correlations. Pairwise correlations, p < 0.0001. The C13 and male specific compounds interaction overlaps at 2.5 males.

**Fig 3:**
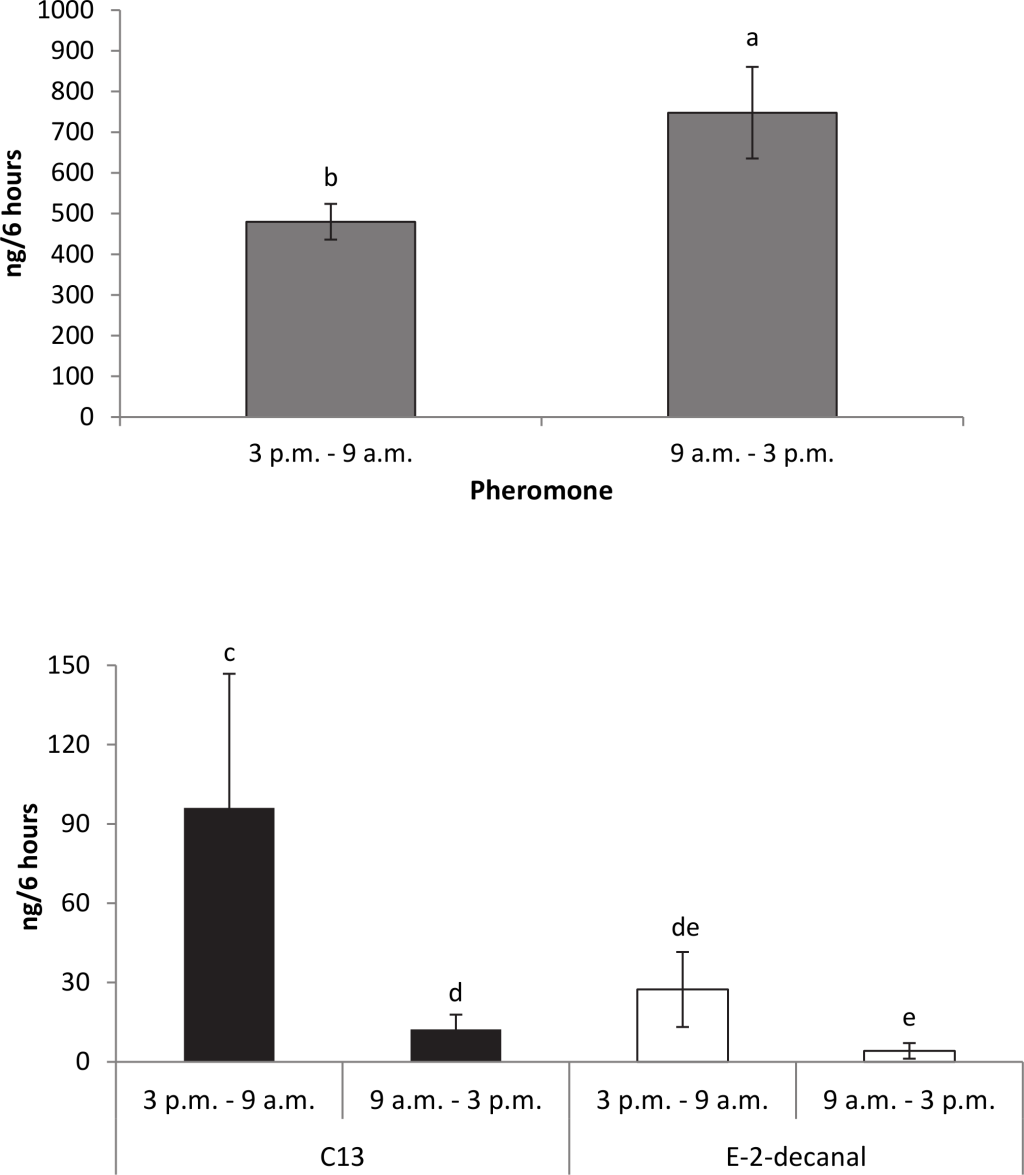
Average amount of pheromone, C13, and *E*-2-decenal in AM and PM collections, standardized by # of hours. Single male *H*. *halys* release more pheromone in the morning/early afternoon than in the evening/overnight, while C13 was released in greater amounts in the evening/overnight. ANOVA: Log (Compound) = Time of day. For pheromone, F_1,209_−35.368, p <0.0001; C13, F_1,209_−5.991, p = 0.0152; *E*-2-decenal, F_1,209_−2.919, p = 0.089. For whole model: Log (Compound) = Time of day, Compound, Time of day*Compound, F_5,629_−151.061, p <0.0001; Time of day F-34.531, p <0.0001; Compound F-350.496, p <0.0001; Time of day *Compound F-8.392, p = 0.001 (n = 7 males, 210 time periods).

Compound emission varied by time of day (Fig 3). Single males released more pheromone in the morning/early afternoon than in the evening/overnight, while C13 was released in greater amounts in the evening/overnight. Amount of compound per time period was standardized for number of hours collected. ANOVA: Log (Compound) = Time of day. For pheromone, F_1,209_−35.368, p <0.0001; C13, F_1,209_−5.991, p = 0.0152; *E*-2-decenal, F_1,209_−2.919, p = 0.089 (n = 7 males, 210 time periods).

**Fig 4:**
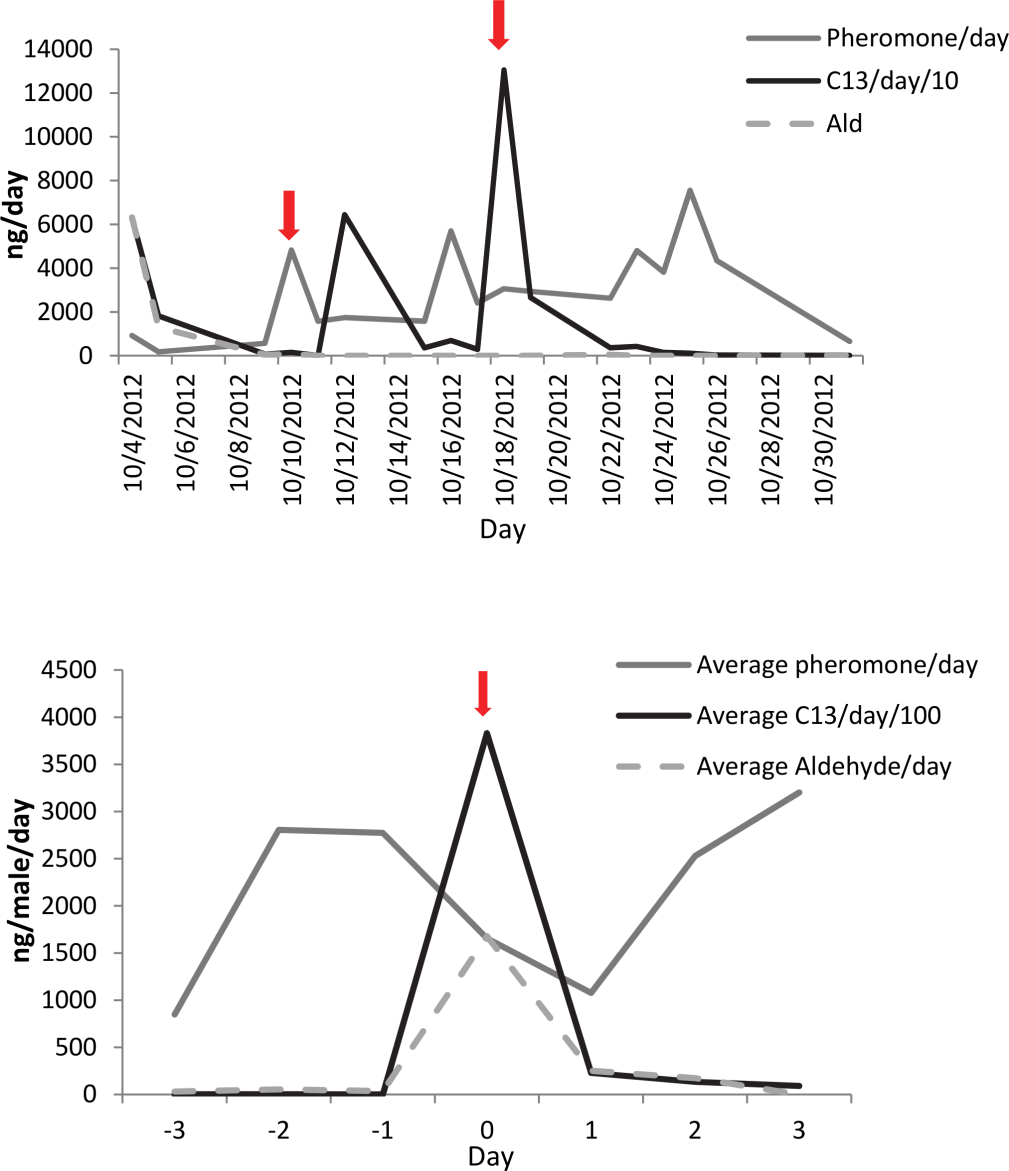
C13 effect on pheromone emission for one male. (A) Arrows indicate date that C13 was added to aeration jars (20 μL on 10/11/12, 30 μL on 10/17/12). (B) Average of single male pheromone emission with application of 20 μL synthetic C13 added to aeration chambers on day 0 (n = 10).

**Fig 5:**
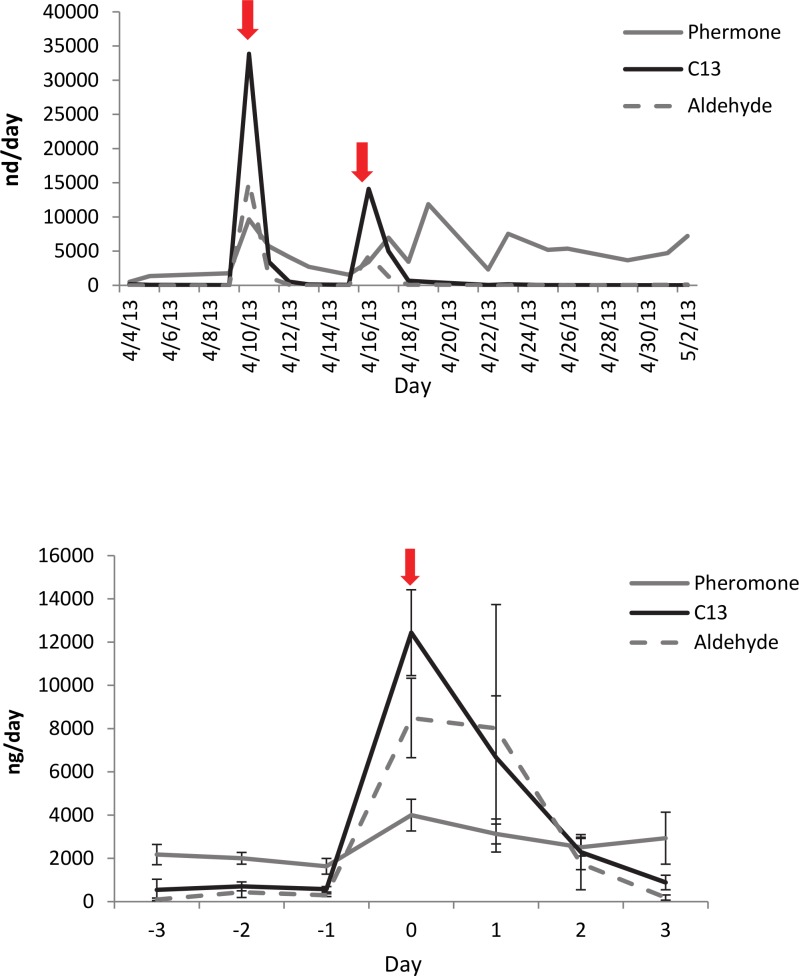
C13 and aldehyde effect on pheromone emission for one male. (A) Arrows indicated date that C13 and aldehyde were added to aeration jars (20 μL C13:Ald at 10:1). (B) Average of single male pheromone emission with application of 20 μL synthetic C13:Ald at 10:1 ratio, added to aeration chambers on day 0 (n = 6).

Synthetic C13 reduces pheromone emission in single males. Fig 4A is an example of C13 effect on pheromone emission for one male. The arrows indicate the date that C13 was added to aeration jars (20 μL on 10/11/12, 30 μL on 10/17/12). Fig 4B shows the average of single male pheromone emission with the application of 20 μL synthetic C13 added to aeration chambers on Day 0 (n = 10). Synthetic C13 + Aldehyde did not reduce pheromone emission in single males. Fig 5A is an example of the effect of 10:1 C13:Aldehyde (20 μL at each arrow). Fig 5B shows the average single male pheromone emission with the application of 20 μL C13 + Aldehyde on Day 0 (n = 6).

### Y-tube Bioassays

**Fig 6:**
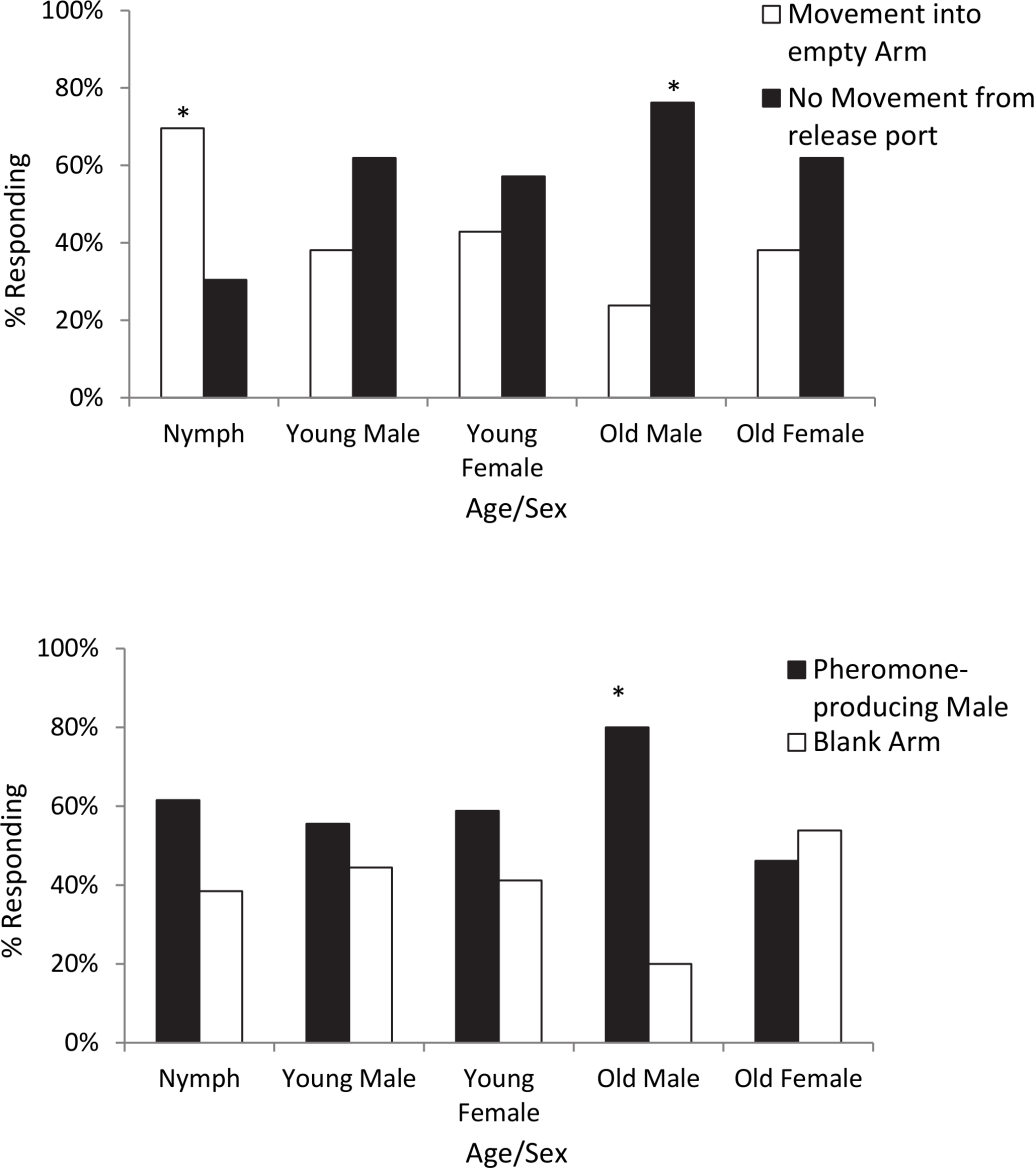
Y-tube bioassay. (A) *H*. *halys* movement in empty Y-tube. Nymphs (3^rd^ stage) are likely to walk in an empty bioassay chamber (positive responders), thus causing a Type II Error. Old males (over 13 days old) are more likely to sit at the entry port to the bioassay chamber when no odors are present (non-responders). Other adults are also more likely to sit in the release chamber than walk to an empty arm of the y-tube, although this is not statistically significant. Nominal Logistic Regression for all age groups in blank chamber: χ^2^–10.32, p = 0.04, DF 4, 101 (n = 21–23). *Denotes p < .05 for age group. (B) *H*. *halys* attraction to live males in empty Y-tube. Old Males (over 13 days old) are significantly attracted to pheromone-emitting males. Nymph: χ^2^–0.70, p = 0.40, n = 13; Young Adult Male: χ^2^–0.22, p = 0.63, n = 18; Young Adult Female: χ^2^–0.53, p = 0.47, n = 17; Old Adult Male: χ^2^–5.78; p = 0.02*, n = 15; Old Adult Female: χ^2^–0.08, p = 0.78, n = 13. Nominal Logistic Regression for all Ages/Sexes: χ^2^–3.92, p = 0.42, DF-4, 76 *Denotes p < 0.05 for age group (likelihood ratio test).

As a control, we tested the likelihood that each age group would move in an empty Y-tube (Fig 6A). For Nymphs: χ^2^–3.62, p = 0.05, n = 23; Young Males: χ^2^–0.43, p = 0.51, n = 21; Young Female: χ^2^–0.43, p = 0.51, n = 21; Old Male: χ^2^–6.06, p = 0.01, n = 21; Old Female: χ^2^–1.20, p = 0.27, n = 21. For the whole model, χ^2^–10.32, p = 0.04, DF 4, 101. Thus, nymphs are very likely to move randomly in the Y-tube and data should be interpreted with caution. Adults, especially males over 13 days into the adult life stage, are more likely to stay in the release chamber when no odor stimuli are present.

Each age group (nymphs, young adults, and old adults) was tested for attraction to pheromone-producing adult males. Twenty to 25 individuals were tested for each age. Percentage of non-responders did not differ by age group (χ^2^–104.38, p = 0.29, DF-4, 93); these data were not included in statistical analysis. Old adult males were the only group significantly attracted to live males in the Y-tube (Fig 6B). Nymphs: χ^2^–0.70, p = 0.40, n = 13; Young adult males: χ^2^–0.22, p = 0.63, n = 18; Young adult females: χ^2^–0.53, p = 0.47, n = 17; Old adult males: χ^2^–5.78, p = 0.02, n = 15; Old adult females: χ ^2^–0.08, p = 0.78, n = 13. Nominal Logistic Regression for all Ages/Sexes: χ^2^–3.92, p = 0.42, DF-4, 76.

## Discussion

*Halyomorpha halys* exhibits similar seasonal behaviors in the U.S. as in its native range in Asia [1]. Adults emerge from overwintering sites in April and begin mating and ovipositing two weeks later [1]. There are five nymphal stages throughout the summer months leading to the next generation adults. In Japan, adults reach sexual maturity around late-September [1,16]. *H*. *halys* reach sexually maturity at 14–15 days after imaginal ecdysis [16]. Early reports of *H*. *halys* life cycles in the mid-Atlantic U.S. reported a single generation [4] in eastern Pennsylvania; however, Leskey et al. recorded bivoltinism in Kearneysville, WV [5].

The average start age for pheromone emission for laboratory-reared *H*. *halys* was 12.71 days. Male *H*. *halys* release pheromone, C13, and *E*-2-decenal at different times during the day, with pheromone being higher from 9 a.m.to 3 p.m., and C13 and *E*-2-decenal being higher in evening hours and overnight, suggesting that insect activity in natural settings may be primarily during the photophase. In other Pentatomidae, mating and feeding periodicity has also been shown to be related to diurnal emissions of aggregation pheromones [17,18].

There are many reports on the circadian rhythm in sex pheromone release for Lepidoptera and Heteroptera [19–22]. However, our data also show that the male-produced pheromone is released in a cyclic spiking pattern every three days, which has not been reported. This could be due to saturation of feedback receptor neurons [23] within the aeration jars or physiological limitations in the production process. C13 is more volatile than the pheromone, and it is possible that this could decrease antennal receptor cell response. This has been observed in moth antennae where compounds with higher volatility impacted electrophysiology and reception [24]. C13 and *E*-2-decenal are released in a less cyclic pattern with an average release time of 6–7 days, and the total amounts of both increase with male density. We showed that synthetic C13 decreases pheromone production, but the absence of repetitive C13 peaks suggests that this compound is not the only factor affecting pheromone emission. Thus, it likely that male *H*. *halys* are sensitive to the dose of ambient pheromone as well. Our studies suggest that male *H*. *halys* do not decrease pheromone production in response to C13 + Aldehyde.

Male *H*. *halys* over 13 days into the adult stage (when pheromone production starts) were significantly attracted to other live males in Y-tube bioassays. While we monitored males daily for high pheromone emission before using them as lures, it is possible that the disturbance of being moved to the Y-tube affected their pheromone release at the time of bioassay. Third stage nymphs exhibited an attractiveness (60–65%) to live males, although this was not statistically significant. Females less than 7 days into the adult stage also showed a similar trend (60%) for movement towards live males. Interestingly, females over 13 days into their adult stage chose the blank control over the odor source. These females were often observed walking to the split in the Y-tube, tapping for 1–5 minutes, and moving into the control arm. Females of the family Pentatomidae have been reported to use bioacoustic signaling in a number of species [25], so it seems that additional feedback stimuli are needed for female *H*. *halys* to complete movement towards an odor stimuli. This should be taken into consideration if this odor blend is used for monitoring wild populations [2].

Our studies show that *H*. *halys* male-produced pheromone emission varies by time of day and density of male conspecifics. Tridecane may be produced in greater quantities at night because it is always proportional with *E*-2-decanal production. Pheromone release is greater during the morning and afternoon hours when aggregation is at its highest. It is possible that conspecifics spread out in the evening hours and overnight. Although live males were only significantly attractive to adult males over 13 days of age in Y-tube assays, it is likely that this pheromone plays a role in aggregating all age groups. Similarly, the presence of C13 and *E*-2-decenal in females and nymphs may play a role in reducing pheromone emission in aggregated groups.

## References

[1] Hoebeke ER, Carter ME (2003) *Halyomorpha halys* (Stal) (Heteroptera: Pentatomidae): a polyphagous plant pest from Asia newly detected in North America. Proceedings of the Entomological Society of Washington 105: 225–237.

[2] Aldrich JR, Khrimian A, Chen X, Camp MJ (2009) Semiochemically based monitoring of the invasion of the brown marmorated stink bug and unexpected attraction of the native green stink bug (Heteroptera: Pentatomidae) in Maryland. Florida Entomologist 92: 483–491.

[3] Leskey TC, Hamilton GC, Nielsen AL, Polk DF, Rodriguez-Saona C, Bergh JC, et al (2012) Pest status of the brown marmorated stink bug, *Halyomorpha halys* (Stål), in the USA. Outlooks on Pest Management 23: 218–266.

[4] Nielsen AL, Hamilton GC (2009) Life history of the invasive species *Halyomorpha halys* (Hemiptera: Pentatomidae) in northeastern United States. Annals of the Entomological Society of America 102: 608–616.

[5] Leskey TC, Short BD, Butler BB, Wright SE (2012) Impact of the invasive brown marmorated stink bug, *Halyomorpha halys* (Stål) in mid-Atlantic tree fruit orchards in the United States: case studies of commercial management. Psyche 2012. 10.1155/2012/535062

[6] Leskey TC, Wright SE, Short BD, Khrimian A (2012) Development of behaviorally based monitoring tools for the brown marmorated stink bug, *Halyomorpha halys* (Stål) (Heteroptera: Pentatomidae) in commercial tree fruit orchards. Journal of Entomological Science 47: 76–85.

[7] Nielsen AL, Shearer PW, Hamilton GC (2008) Toxicity of insecticides to *Halyomorpha halys* (Hemiptera: Pentatomidae) using glass-vial bioassays. Journal of Economic Entomology 101: 1439–1442. 18767758 10.1603/0022-0493(2008)101[1439:toithh]2.0.co;2

[8] McBrien HL, Millar JG, Gottlieb L, Chen X, Rice RE (2001) Male-produced sex attractant pheromone of the green stink bug, *Acrosternum hilare* (Say). Journal of Chemical Ecology 27: 1821–1839. 11545373 10.1023/a:1010460709535

[9] Aldrich JR (1988) Chemical ecology of the Heteroptera. Annual Review of Entomology 33: 211–238. 10.1146/annurev-ento-010715-023507 26982440

[10] Ota D, Cokl A (1991) Mate location in the Southern green stink bug, *Nezara viridula* (Heteroptera: Pentatomidae), mediated through substrate-borne signals on Ivy. Journal of Insect Behavior 4: 441–447.

[11] Zunic A, Cokl A, Doberlet MV, Millar JG (2008) Communication with signals produced by Abdominal Vibration, Tremulation, and Percussion in *Podisus maculiventris* (Heteroptera: Pentatomidae). Annals of the Entomological Society of America 101: 1169–1178.

[12] Aldrich JR, Khrimian A, Camp MJ (2007) Methyl 2,4,6-decatrienoates attract stink bugs and tachinid parasitoids. Journal of Chemical Ecology 33: 801–815. 17334917 10.1007/s10886-007-9270-9

[13] Khrimian A, Zhang A, Weber DC, Ho H-Y, Aldrich JR, Vermillion KE, et al (2014) Discovery of the aggregation pheromone of the brown marmorated stink bug (*Halyomorpha halys*) through the creation of stereoisomeric libraries of 1-bisabolen-3-ols. Journal of Natural Products 77: 1708–1717. 10.1021/np5003753 24963992

[14] Zhang A, Facundo HT, Robbins PS, Linn CE Jr., Hanula JL, Villani MG, et al (1994) Identification and synthesis of female sex pheromone of Oriental beetle, *Anomala orientalis* (Coleoptera: Scarabaeidae). Journal of Chemical Ecology 20: 2415–2427. 10.1007/BF02033210 24242814

[15] Zhang A, Amalin D, Shirali S, Serrano MS, Franqui RA, Oliver JE, et al (2004) Sex pheromone of the pink hibiscus mealybug, *Maconellicoccus hirsutus*, contains an unusual cyclobutanoid monoterpene. Proceedings of the National Academy of Sciences of the United States of America 101: 9601–9606. 15197282 10.1073/pnas.0401298101 PMC470721

[16] Kawada H, Kitamura C (1983) The reproductive behavior of the brown marmorated stink bug, *Halyomorpha mista* Uhler (Heteroptera: Pentatomidae) .1. Observation of mating behavior and multiple copulation. Applied Entomology and Zoology 18: 234–242.

[17] Krupke CH, Jones VP, Brunner JF (2006) Diel periodicity of *Euschistus conspersus* (Heteroptera: Pentatomidae) aggregation, mating, and feeding. Annals of the Entomological Society of America 99: 169–174.

[18] Moriya S, Shiga M (1984) Attraction of the male brown-winged green bug, *Plautia stali* Scott for males and females of the same species. Applied Entomology and Zoology 19: 317–322.

[19] Baker TC, Carde RT (1979) Endogenous & exogenous factors affecting periodicities of female calling and male sex pheromone response in *Grapholitha molesta* (Busck). Journal of Insect Physiology 25: 943–950.

[20] Silvegren G, Lofstedt C, Rosen WQ (2005) Circadian mating activity and effect of pheromone pre-exposure on pheromone response rhythms in the moth *Spodoptera littoralis* . Journal of Insect Physiology 51: 277–286. 10.1016/j.jinsphys.2004.11.013 15749110

[21] Levi-Zada A, David M, Fefer D, Seplyarsky V, Sadowsky A, Dobrinin S, et al (2014) Circadian release of male-specific components of the greater date moth, *Aphomia* (Arenipses) *Sabella*, using sequential SPME/GC/MS analysis. Journal of Chemical Ecology 40: 236–243. 10.1007/s10886-014-0391-7 24567046

[22] Zahn DK, Moreira JA, Millar JG (2008) Identification, synthesis, and bioassay of a male-specific aggregation pheromone from the harlequin bug, *Murgantia histrionica* . Journal of Chemical Ecology 34: 238–251. 10.1007/s10886-007-9415-x 18204884

[23] Ryan MF (2002) Insect Chemoreception: Fundamental and Applied. Dordrecht: Kluwer Academic Publishers.

[24] Prestwich GD, Sun WC, Mayer MS, Dickens JC (1990) Perfluorinated moth pheromones: Synthesis and electrophysiological activity. Journal of Chemical Ecology 16: 1761–1778. 10.1007/BF01020493 24263983

[25] Cokl A, Doberlet MV (2003) Communication with substrate-borne signals in small plant-dwelling insects. Annual Review of Entomology 48: 29–50. 12414736 10.1146/annurev.ento.48.091801.112605

